# An individualised versus a conventional pneumoperitoneum pressure strategy during colorectal laparoscopic surgery: rationale and study protocol for a multicentre randomised clinical study

**DOI:** 10.1186/s13063-019-3255-1

**Published:** 2019-04-03

**Authors:** O. Diaz-Cambronero, G. Mazzinari, C. L. Errando, M. J. Schultz, B. Flor Lorente, N. García-Gregorio, M. Vila Montañés, Daniel Robles-Hernández, L. E. Olmedilla Arnal, A. Martín-De-Pablos, A. Marqués Marí, M. P. Argente Navarro, for the IPPCollapse-II study group, Oscar Díaz-Cambronero, Blas Flor-Lorente, Guido Mazzinari, María Vila-Montañés, Nuria García-Gregorio, Maria Jose Alberola-Estellés, Begoña Ayas-Montero, Salome Matoses-Jaén, Sandra Verdeguer, Anabel Marqués Marí, Jose Miguel Alonso Íñigo, Josep Balaguer Domenech, Marisol Echeverri Velez, David Cuesta-Frau, Maria Pilar Argente-Navarro, Salvador Pous, Cristina Ballester, Matteo Frasson, Alvaro García-Granero, Carlos Cerdán-Santacruz, Eduardo García-Granero, Luis Sánchez-Guillén, Daniel Robles-Hernández, David Boquera-Albert, David Casado-Rodrigo, Rebeca Cosa-Rodríguez, Luis Enrique Olmedilla-Arnal, Marcos Rodríguez-Martín, Jaime Zorrilla-Ortúzar, José María Pérez-Peña, Angel Martín-de-Pablos, Javier Valdés-Hernández, Juan Carlos Gómez-Rosado, Pino Heredia-Pérez, Juan Cintas-Catena, Fernando Flor-Parra, Marcus J. Schultz, Carlos Luis Errando Oyonarte

**Affiliations:** 1 Department of Anaesthesiology, Hospital Universitari i Politècnic La Fe, Valencia, Spain; 2 Perioperative Medicine Research Group, Instituto de Investigación Sanitaria La Fe (IIS laFe), Avinguda de Fernando Abril Martorell 106, 46026 Valencia, Spain; 3 SCReN-IIS La Fe, PT17/0017/0035, Spanish Clinical Research Network (SCReN), Valencia,, Spain; 4 Department of Anaesthesiology, Hospital Universitari i Politecnic la Fe , Valencia, Spain; 5 Department of Anaesthesiology, Consorcio Hospital General Universitario de Valencia, Valencia, Spain; 6 Department of Intensive Care & Laboratory of Experimental Intensive Care and Anesthesiology (L·E·I·C·A), Academic Medical Center, Amsterdam, The Netherlands; 7 Mahidol Oxford Tropical Medicine Research Unit (MORU), Mahidol University, Bangkok, Thailand; 8 Department of Colorectal Surgery, Hospital Universitari i Politècnic La Fe, Valencia, Spain; 9 Department of Anaesthesiology, Hospital General Universitario de Castellón, Castellón, Spain; 10 Department of Anaesthesiology, Hospital General Universitario Gregorio Marañón, Madrid, Spain; 11 Department of Anaesthesiology, Hospital Universitario Virgen Macarena, Sevilla, Spain

**Keywords:** Abdominal laparoscopy, Colorectal surgery, Pneumoperitoneum pressure, Outcome, Post-operative Quality of Recovery Scale (PQRS), Postoperative complications, Safety

## Abstract

**Background:**

A recent study shows that a multifaceted strategy using an individualised intra-abdominal pressure titration strategy during colorectal laparoscopic surgery results in an acceptable workspace at low intra-abdominal pressure in most patients. The multifaceted strategy, focused on lower to individualised intra-abdominal pressures, includes prestretching the abdominal wall during initial insufflation, deep neuromuscular blockade, low tidal volume ventilation settings and a modified lithotomy position. The study presented here tests the hypothesis that this strategy improves outcomes of patients scheduled for colorectal laparoscopic surgery.

**Methods:**

The Individualized Pneumoperitoneum Pressure in Colorectal Laparoscopic Surgery versus Standard Therapy (IPPCollapse-II) study is a multicentre, two-arm, parallel-group, single-blinded randomised 1:1 clinical study that runs in four academic hospitals in Spain. Patients scheduled for colorectal laparoscopic surgery with American Society of Anesthesiologists classification I to III who are aged > 18 years and are without cognitive deficits are randomised to an individualised pneumoperitoneum pressure strategy (the intervention group) or to a conventional pneumoperitoneum pressure strategy (the control group). The primary outcome is recovery assessed with the Post-operative Quality of Recovery Scale (PQRS) at postoperative day 1. Secondary outcomes include PQRS score in the post anaesthesia care unit and at postoperative day 3, postoperative complications until postoperative day 28, hospital length of stay and process-related outcomes.

**Discussion:**

The IPPCollapse-II study will be the first randomised clinical study that assesses the impact of an individualised pneumoperitoneum pressure strategy focused on working with the lowest intra-abdominal pressure during colorectal laparoscopic surgery on relevant patient-centred outcomes. The results of this large study, to be disseminated through conference presentations and publications in international peer-reviewed journals, are of ultimate importance for optimising the care and safety of laparoscopic abdominal surgery. Selection of patient-reported outcomes as the primary outcome of this study facilitates the translation into clinical practice. Access to source data will be made available through anonymised datasets upon request and after agreement of the Steering Committee of the IPPCollapse-II study.

**Trial registration:**

ClinicalTrials.gov, NCT02773173. Registered on 16 May 2016. EudraCT, 2016-001693-15. Registered on 8 August 2016.

**Electronic supplementary material:**

The online version of this article (10.1186/s13063-019-3255-1) contains supplementary material, which is available to authorized users.

## Background

Compared to open surgery, laparoscopic surgery generally results in better outcomes [1, 2]. Compared to open abdominal surgery, a laparoscopic approach during abdominal surgery is associated with less blood loss and fewer needs for blood transfusions [3, 4], faster recovery of bowel function and oral intake resumption [5, 6], fewer analgesic requirements [6, 7] and a shorter length of hospital stay (LOS) [3–8]. Patient-reported outcomes (PROs) are new tools for testing quality of recovery in the postoperative setting, and the Post-operative Quality of Recovery Scale (PQRS) has been successfully tested in previous studies.

A high intraoperative intra-abdominal pressure (IAP) is clearly associated with perioperative morbidity [9–14]. While guidelines for laparoscopic abdominal surgery recommend the lowest possible IAP at which the surgeon has adequate workspace rather than using a predetermined level [15, 16], it remains common practice to use a standard IAP level throughout the surgical procedure, usually between 12 and 15 mmHg and sometimes even higher depending on surgical indication [17]. Interestingly, while the surgical condition depends mainly on the intra-abdominal volume (IAV) and the workspace obtained at a given IAP, the focus during pneumoperitoneum insufflation remains with the applied IAP [18].

Several factors improve the relation between IAP and the obtained surgical workspace, including patient positioning [19], use of neuromuscular blockade [20, 21] and prestretching of the abdominal wall [22]. The previous pivotal and feasibility study, Individualized Pneumoperitoneum Pressure in Colorectal Laparoscopic Surgery I (IPPCollapse-I), showed that combining all these factors with individualised IAP titration resulted in an acceptable workspace at 8 mmHg IAP in 61 out of 78 patients (78%) [23]. The IPPCollapse-II study presented here tests the hypothesis that this individualised pneumoperitoneum pressure strategy improves recovery of PQRS score when compared to a conventional strategy that uses a fixed pneumoperitoneum pressure approach in patients undergoing scheduled colorectal laparoscopic surgical intervention.

## Methods/design

### Study reporting

This report follows the Standard Protocol Items: Recommendations for Interventional Trials and Patient-Reported Outcomes (SPIRIT-PRO) guidelines [24, 25]. Additional file 1 details the IPPCollapse-II SPIRIT checklist.

### Study design

**Fig. 1:**
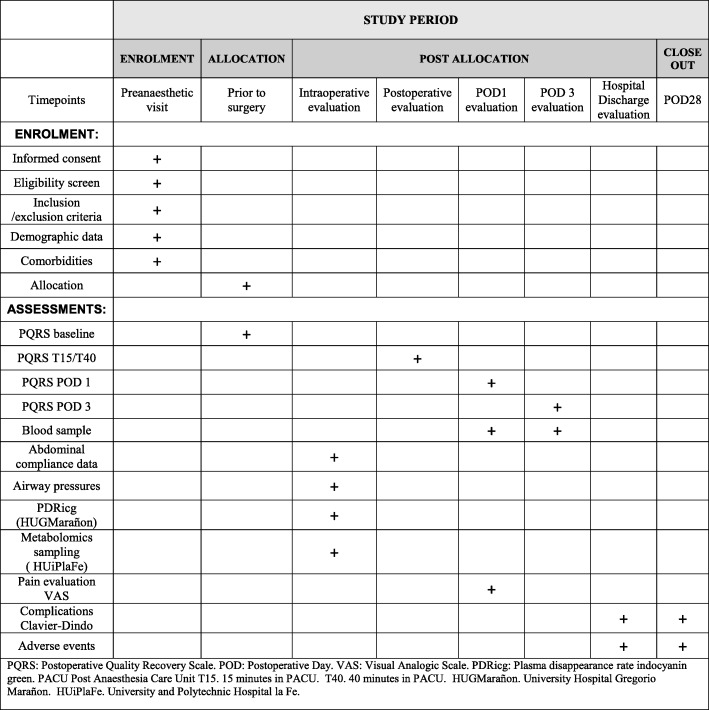
Study timepoints

**Fig. 2:**
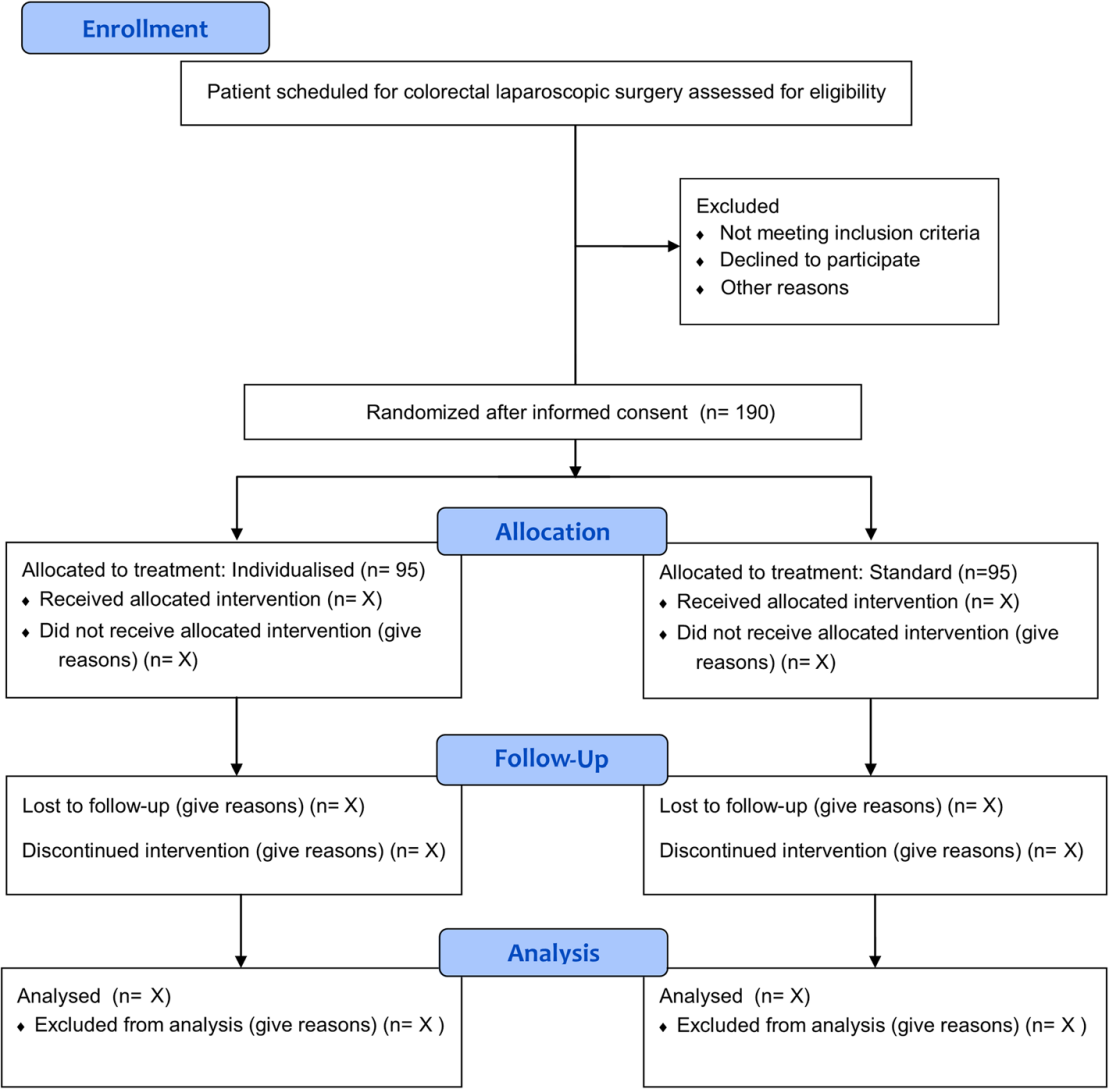
IPPCollapse II flowchart

The IPPCollapse-II study is a multicentre, two-arm, parallel-group, single-blinded randomised clinical study. The enrolment and assesments during the study period are shown in Fig. 1. The study flowchart is shown in Fig. 2.

### Study setting

The IPPCollapse-II study runs in the operating room and surgical wards of four academic hospitals in Spain (detailed in Additional file 2).

### Study population

Patients are eligible for participation if they (1) are scheduled for laparoscopic colorectal surgery, (2) are aged > 18 years, (3) have an American Society of Anesthesiologists (ASA) physical status I to III, and (d) have no cognitive deficits. Exclusion criteria are (1) no written informed consent; (2) emergency or unplanned surgery; (3) pregnancy or breastfeeding; (4) immunologic or neuromuscular diseases; (5) advanced stage of cardiopulmonary, renal or hepatic disease; and (6) allergy to or contraindications for rocuronium or sugammadex.

### Randomisation and blinding

Patients are randomised in a 1:1 ratio to an individualised pneumoperitoneum pressure strategy (the intervention group) or a standard pneumoperitoneum pressure strategy (the control group). Local investigators perform randomisation using a web-based automated randomisation system (Biostatistics Unit of the Health Research Institute La Fe, Valencia, Spain). Randomisation is performed with random block sizes and is stratified per centre. While attending anaesthesiologists are aware of the assigned pneumoperitoneum pressure strategy, the attending surgeons as well as the patients remain unaware of the assigned pneumoperitoneum pressure strategy at all times, i.e. before surgery, during surgery and after surgery. The PQRS is a patient-reported outcome (PRO), meaning that the investigator has little room to cause bias. The pneumoperitoneum insufflator screen is covered by a surgical drape. Study team members, who are not blinded to randomisation, perform postoperative PQRS measurements.

### Standard pneumoperitoneum pressure strategy

The standard strategy consists of the following elements, to be performed in the same order in all patients in the control group: (1) patients are placed in a position according to the surgeon’s preference within a predefined range of Trendelenburg position (0–30°); (2) patients receive moderate neuromuscular blockade with rocuronium, cisatracurium or atracurium throughout surgery to maintain a train of four (TOF) between 2 and 4; and (3) the IAP is set at 12 mmHg throughout surgery. At any time, surgeons can request an IAP increase if the workspace becomes ‘inadequate’; in that case, the IAP is increased in steps of 1 mmHg during 1-min intervals to a maximum of 15 mmHg, but not higher than the level at which the surgical workspace returns to ‘adequate’. Surgeons will be warned if the IAP reaches the predefined upper limit.

Neuromuscular blockade pharmacological reversion is achieved with neostigmine (2.5 mg or 30–50 μg∙kg^− 1^), according to usual care.

### Individualised pneumoperitoneum pressure strategy

The multifaceted individualised pneumoperitoneum strategy consists of the following elements, which will be performed in the same order in all patients in the intervention group: (1) patient position is modified to increase the anteroposterior intra-abdominal space by correcting lumbar lordosis; (2) patients receive deep neuromuscular blockade throughout surgery to maintain a TOF of 0 and a post-tetanic count (PTC) between 1 and 5; (3) the abdominal wall and muscles are prestretched by maintaining an IAP of 15 mmHg for 5 min during the first CO_2_ gas insufflation and insertion of trocars (to achieve this, the CO_2_ gas insufflator will be initially set at 15 mmHg with a flow rate of 3 L∙min^− 1^); and (4) individualised IAP titration is used when the patient is placed in the surgical position (0–30° Trendelenburg); for this, the flow rate is increased to 30 L∙min^− 1^ and the IAP is decreased from 15 to 12 mmHg, and thereafter stepwise to 11, 10, 9 and finally 8 mmHg as long as the attending surgeon keeps an ‘adequate’ workspace. As in the standard pneumoperitoneum pressure group, surgeons can request an IAP increase up to 15 mmHg, which will be performed likewise. Of note, a pressure increment can be requested by the surgeon at all times in both study arms, where it always follows a similar approach; the previous feasibility study showed that an IAP increase was requested only one time in one-fifth of the patients and never twice [23].

Neuromuscular blockade pharmacological reversion at the end of surgery, before tracheal extubation, is achieved with sugammadex 4 mg∙kg^− 1^.

For clarity, the elements of the two group strategies are summarised in Table 1.

**Table 1:** Intervention sequence

Standard pneumoperitoneum pressure strategy (SPP group)	Individualised pneumoperitoneum pressure strategy (IPP group)
1. Trendelenburg (0–30°) placement	1. Trendelenburg (0–30°) + ‘modified lithotomy position’, with flexed hips (between 45 and 90°) and legs raised in padded supports to increase anteroposterior intra-abdominal space
2. Moderate neuromuscular blockade throughout surgery (TOF between 2 and 4)	2. Deep neuromuscular blockade throughout surgery (TOF of 0 and a PTC between 1 and 5)
3. No prestretching of abdominal wall muscles	3. Prestretching of abdominal wall muscles by maintaining an IAP of 15 mmHg for 5 min during the first CO_2_ gas insufflation and insertion of trocars (flow rate at 3 L∙min^− 1^)
4. IAP is set at 12 mmHg throughout surgery	4. IAP down-titration (flow rate at 30 L∙min^− 1^) from 15 to 12 mmHg, and thereafter stepwise to 11, 10, 9 and finally 8 mmHg as long as ‘adequate’ workspace is preserved (by surgeon’s judgement)
5. Surgeons can request an IAP increase if workspace becomes ‘inadequate. IAP is increased in steps of 1 mmHg during 1-min intervals to a maximum of 15 mmHg. Surgeons are warned when upper limit is reached	5. Surgeons can request an IAP increase if workspace becomes ‘inadequate’; IAP is increased in steps of 1 mmHg during 1-min intervals to a maximum of 15 mmHg. Surgeons are warned when upper limit is reached

*TOF* train of four, *IAP* intra-abdominal pressure, *PTC* post-tetanic count

### Standard care

Perioperative management other than the pneumoperitoneum strategy is suggested to follow the Spanish Enhanced Recovery Pathways recommendations (detailed in Additional file 3) [26]. Continuous intraoperative neuromuscular monitoring with acceleromyography (TOF-Watch-SX™, Organon Teknika, Oss, The Netherlands) is used. At the end of surgery, the neuromuscular blockade will be fully reversed to a TOF ratio (TOFr) of at least 0.9 before tracheal extubation. An electronic CO_2_ insufflator (Endoflator™, Karl Storz, Tuttlingen, Germany) will be used for gas insufflation into the abdominal cavity through a paraumbilically placed laparoscopic trocar/Veress needle.

Patients in both groups will be ventilated in a volume-controlled ventilation mode, using a tidal volume of 8 ml/kg predicted ideal body weight, with a 20% inspiratory pause time, and positive end-expiratory pressure set at 5 or 10 mmHg in patients with a body mass index (BMI) < 30 or ≥ 30 kg∙m^− 2^, respectively. Oxygen inspiratory fraction is 0.8 throughout surgery. Respiratory rate is set at 12–15 breaths per minute to maintain normal end-tidal CO_2_ values [27].

### Primary outcome

The primary outcome is the recovery of the PQRS at postoperative day 1 (POD1) (see the subsequent sections for details).

### Secondary outcomes

Secondary outcomes include recovery of PQRS at 15 min (T15) and at 40 min (T40) after arrival in the post anaesthesia care unit (PACU) and in the surgical wards during the morning at postoperative day 3 (POD3). Other secondary clinical outcomes include daily postoperative complications until hospital discharge and at postoperative day 28, hospital length of stay (LOS) and secondary process-related outcomes that include the highest IAP level and IAV at which surgery could be performed, hepatic perfusion during pneumoperitoneum and the ventilatory parameters plateau pressure and driving pressure.

Occurrences of diaphragm and abdominal wall contractions or spontaneous breathing efforts and coughing during surgery are collected and compared between the two study groups.

### Substudies

The IPPCollapse-II study has three substudies (see the detailed description in Additional file 4):

1. Levels of biomarkers (neutrophil-lymphocyte ratio, C-reactive protein, interleukin-6 and procalcitonin) are measured in peripheral venous blood samples obtained before surgery and at POD1 and POD3 and compared between the two study groups. For this substudy, blood samples are obtained in all participating centres.
2. Untargeted metabolomic analysis is performed on peripheral venous blood samples and peritoneal tissue, both obtained after initial insufflation of pneumoperitoneum and at the end of the procedure. This substudy includes the first 10 patients in the Hospital Universitari i Politècnic La Fe, Valencia, Spain.
3. Plasma disappearance rate of indocyanine green (PDR_ICG_) after intravenous ICG injection is measured to evaluate hepatic perfusion during pneumoperitoneum as a marker of liver function [28]. This substudy runs only at the University Hospital Gregorio Marañon, Madrid, Spain.

### Post-operative Quality of Recovery Scale

The PQRS is a validated multidimensional PRO tool [29–31] designed to assess patients’ recovery to baseline status in the postoperative period (www.postopqrs.com). In every patient a baseline measurement of PQRS is performed prior to surgery. After surgery, the measurement of the PQRS is repeated at 15 min (T15) and at 40 min (T40) after arrival in the PACU, as well as in the ward on the morning of POD1 and POD3. The PQRS is a verbal survey tool that depicts recovery in the following five domains: physiologic, nociceptive, emotive, functional, cognitive, and also collects overall patient perspective. Each of these domains is assessed with multiple items on an ordinal scale and compared with baseline to evaluate recovery (see Table 2 for details). Recovery is a dichotomised outcome defined by a return to at least baseline values or better at each of the postoperative measurement time points. Overall recovery requires recovery in all domains being assessed, and failure in any domain results in failure of overall recovery.

**Table 2:** Post-operative Quality of Recovery Scale (PQRS)

Domain	Variable	Score	Baseline	T15	T40	POD1	POD3
Physiologic	Blood pressure	1–3	+	+	+	+	+
Physiologic	Heart rate	1–3	+	+	+	+	+
Physiologic	Temperature	1–3	+	+	+	+	+
Physiologic	Respiration	1–3	+	+	+	+	+
Physiologic	SpO2	1–3	+	+	+	+	+
Physiologic	Airway	1–3	+	+	+	+	+
Physiologic	Agitation	1–3	+	+	+	+	+
Physiologic	Consciousness	1–3	+	+	+	+	+
Physiologic	Activity on command	1–3	+	+	+	+	+
Nociceptive	Pain	1–5 Likert	+	+	+	+	+
Nociceptive	PONV	1–5 Likert	+	+	+	+	+
Emotional	Sadness/depression	1–5 Likert	+	+	+	+	+
Emotional	Anxiety/nervousness	1–5 Likert	+	+	+	+	+
Functional	Stand	1–3	+	–	–	+	+
Functional	Walk	1–3	+	–	–	+	+
Functional	Eat/drink	1–3	+	–	–	+	+
Functional	Get dressed	1–3	+	–	–	+	+
Cognitive	Name, city and DOB	TF 0	+	–	–	+	+
Cognitive	Numbers forward	TF 2	+	–	–	+	+
Cognitive	Numbers backwards	TF 1	+	–	–	+	+
Cognitive	Word task: list	TF 3	+	–	–	+	+
Cognitive	Executive memory	TF 3	+	–	–	+	+
Overall patient perspective	Ability to work	1–5	–	–	–	–	+
Overall patient perspective	Ability to perform ADLs	1–5	–	–	–	–	+
Overall patient perspective	Clarity of thought	1–5	–	–	–	–	+
Overall patient perspective	Satisfaction anaesthesia care	1–5	–	–	–	–	+

Online scale to assess multiple domains of postoperative recovery over time. Timeline: T15–15 min in PACU, T40–40 min in PACU Scoring: Physiologic 1–3; Nociceptive/emotional 1–5, Likert rating scale using a faces pictorial display; Functional: Scored as 3 easily, 2 with difficulty, 1 not at all; Cognitive: Performance variability tolerance factor (TF) is applied. Participants not included in subsequent analysis if baseline scores are equal to or less than the TF *PACU* post-anaesthesia care unit, *POD1* postoperative day 1, *POD3* postoperative day 3, *SpO2* blood oxygen saturation, *PONV* postoperative nausea and vomiting, *DOB* date of birth, *ADL* activity of daily living

### Definitions

The IAP will be recorded as read from the gas insufflator device. In the intervention group the ‘individualised IAP’ is defined as the highest IAP needed to obtain and maintain an adequate workspace until completion of surgery. The IAV is calculated by linear interpolation from the patient’s IAP/IAV curve obtained during initial pneumoperitoneum insufflation matching to the IAP at which surgery is performed.

‘Adequate’ workspace is defined as the intra-abdominal workspace sufficient to perform the surgical procedure with no need for corrective manoeuvres (i.e. IAP increase) as judged by the attending surgeon, who remains blinded for the actual IAP. Consequently, ‘inadequate’ workspace is defined as an intra-abdominal workspace insufficient to perform the surgical procedure with the need for corrective manoeuvres (i.e. IAP increase).

Definitions of the various postoperative complications recorded are in accordance with the current European standards for perioperative outcomes (Table 3) [32]. Severity of postoperative complications is evaluated using Clavien-Dindo grading (Table 4) [33].

**Table 3:** Classification of postoperative complications

1. Acute kidney damage	
2. Acute respiratory distress syndrome (ARDS)	
3. Suture dehiscence	
4. Arrhythmia	
5. Cardiac arrest	
6. Cardiogenic pulmonary edema	
7. Deep vein thrombosis	
8. Postoperative delirium	
9. Gastrointestinal bleeding	
10. Infection	
11. Bacteraemia	
12. Myocardial infarction	
13. Myocardial injury after non-cardiac surgery	
14. Pneumonia	
15. Paralytic ileus	
16. Postoperative haemorrhage	
17. Pulmonary embolism	
18. Cerebrovascular accident	
19. Infection of surgical wound (superficial)	
20. Infection of surgical wound (deep)	
21. Infection of surgical (organ) wound	
22. Urinary tract infection	
Postoperative pulmonary complications:	
1. Respiratory infection	
2. Respiratory failure	
3. Pleural effusion	
4. Atelectasis	
5. Pneumothorax	
6. Bronchospasm	
7. Pneumonia due to aspiration

**Table 4:** Severity grade by Clavien-Dindo definition

Grade I	Any deviation from the normal postoperative course without the need for pharmacological treatment or surgical, endoscopic or radiological interventions Allowed therapeutic regimens are drugs as antiemetic, antipyretics, analgesics, diuretics and electrolytes and physiotherapy. This grade also includes wound infections opened at the bedside
Grade II	Requiring pharmacological treatment with drugs other than such allowed for Grade I complications Blood transfusions and total parenteral nutrition are also included
Grade III	Requiring surgical, endoscopic or radiological intervention
- IIIa	Intervention not under general anaesthesia
- IIIb	Intervention under general anaesthesia
Grade IV	Life-threatening complication (including CNS complications)^a^ requiring IC/ICU management
- IVa	Single organ dysfunction (including dialysis)
- IVb	Multiorgan dysfunction
Grade V	Death of a patient
Suffix ’d’	If the patient suffers from a complication at the time of discharge, the suffix ’d’ (for ‘disability’) is added to the respective grade of complication. This label indicates the need for a follow-up to fully evaluate the complication

^a^Brain haemorrhage, ischaemic stroke, subarrachnoidal bleeding, but excluding transient ischaemic attacks (TIAs)

*IC* intermediate care, *ICU* intensive care unit, *CNS* central nervous system

Respiratory system driving pressure (ΔP_rs_) is calculated by subtracting positive end-expiratory pressure (PEEP) from Pressure plateau (Pplat) [34]. Perioperative safety issues are recorded during the surgery and are related to involuntary patient movements, and defined as diaphragm or abdominal wall contractions or spontaneous breathing efforts or coughing during anaesthesia.

Hospital LOS is defined as hospital discharge date minus hospital admission date.

### Data to be collected

Before anaesthesia demographic data will be collected including age (years), gender, body height (centimetres) and body weight (kilograms), BMI (kilograms per metre squared), ASA physical status score, comorbidities, number of previous abdominal surgeries and number of previous laparoscopic surgeries and PQRS score.

During anaesthesia the following data will be obtained: levels of IAPs at which surgery is performed (mmHg) in both groups; proportion of patients who needed a pressure increment to achieve acceptable surgical workspace; IAV at start of pneumoperitoneum (litres); coughing and spontaneous movements (yes/no); type of surgery and oncologic status; duration of surgery (minutes); duration of anaesthesia (minutes); proportion of patients who needed conversion from laparoscopic to open surgery and the reason for it (only if applicable); ventilation data including PEEP, plateau pressure and respiratory driving pressure (ΔP_rs_) (all in centimetres of H_2_O pressure) before pneumoperitoneum generation and during initial IAP titration until a stable level of IAP is reached in both groups; type and dose of neuromuscular blocking agent (milligrams); type and dose of neuromuscular blocking reversal agent (milligrams); total opioid requirement during the first 24 h if used (milligrams); and PDR_ICG_ in the stable pneumoperitoneum phase.

Directly after anaesthesia, in the PACU the following will be obtained: PQRS score at 15 and 40 min after PACU admission and on POD1 and POD3; PQRS score in the morning and peripheral venous blood samples for determination levels of biomarkers.

On all postoperative days until hospital discharge and at day 28 the occurrence of postoperative complications and location will be noted.

### Analysis plan

The statistical analysis plan (SAP) is specified before enrolment of the first patient. In the absence of studies assessing differences in recovery, based on intraoperative IAP management during laparoscopic colorectal surgery, we performed the sample size calculation assuming an odds ratio of 2.65 (equivalent to a difference of 0.5 unit in the logit scale) between groups in the recovery of physiologic PQRS score. It was estimated that a sample size of 170 patients is required to achieve 80% power at a significance level of alpha = 0.05. All reasons for dropouts, expected to be as low as 10%, will be collected and reported. Conversion to open surgery was the main reason for dropouts in the previous study. We will recruit a total of 190 patients to compensate for potential losses.

All analyses will be performed with R software (R Foundation for Statistical Computing, Vienna, Austria). Data will be expressed as the mean (standard deviation, SD) or median (interquartile range, IQR) for continuous variables depending on their distribution (normality will be checked with the Shapiro-Wilks test) and by counts and proportions for categorical variables. The 95% confidence intervals will be calculated for each of the estimated percentiles. The statistical significance level will be set at *P* < 0.05.

The analysis of the primary endpoint follows the intention-to-treat principle. The difference between the recovery PQRS score between groups, the primary outcome on POD1, will be assessed by mixed ordinal logistic regression introducing the patient as random factor, and age, weight, BMI and sex as covariables. The differences in Clavien-Dindo grading of postoperative complications will be assessed by ordinal regression.

For IAV calculation the relationship between IAP and the insufflated volume of CO_2_ will be determined for each patient during initial pneumoperitoneum insufflation. The relationship between IAP and IAV was analysed by linear interpolation from the individual IAP/IAV curves to determine the actual IAV at which surgery is performed. The IAP before CO_2_ gas insufflation was considered the basal IAP or IAP at volume zero, and it was estimated by fitting multiadaptive linear regression splines to the IAV and IAP relationship.

Differences in continuous variables between groups (IAP, IAV, LOS, inflammatory biomarkers) will be assessed by linear regression or with the Mann-Whitney *U* test (if the normal distribution assumption is rejected by the Shapiro-Wilks test). Differences in ΔP_rs_ between groups will be assessed by linear regression. A multivariable model introducing BMI, previous laparoscopic surgery and age will be fitted for predictive purposes.

Differences in the plasma disappearance rate of ICG are assessed by beta regression. Occurrences of cough or spontaneous movements during anaesthesia are assessed by logistic regression.

The relationship between IAP and IAV will analysed by linear interpolation from the individual IAP/IAV curves. The IAP before CO_2_ gas insufflation (IAP at volume zero) will be estimated by fitting multiadaptive linear regression splines to the IAV and IAP relationship. If a variable has a frequency of missing data > 5%, data will be imputed by the multiple imputation method.

As there is no ethically unacceptable risk related to the primary outcome analysed, there will be no planned interim analysis.

### Adverse events

All adverse events (AEs) or serious adverse events (SAEs), related to the study medication or not, will be followed up by the investigators and documented in the electronic case report form (eCRF) up to 28 days after the end of the intervention period. All SAEs will be notified to the Steering Committee and promoter of the study as soon as the researcher has knowledge of the SAEs, but not more than 24 h after the researcher becomes aware of the event.

### Auditing

Sites may be subject to audits, independent ethics committee (IEC)/Institutional Review Board (IRB) review and regulatory inspection(s). Local investigators will provide direct access to the source data documents (see Additional file 4 for full details).

### Ethics and dissemination

The study will be carried out according to a protocol reviewed and approved at a national level by the IRB of Hospital Universitari i Politècnic La Fe, Valencia, Spain, and Agencia Española del Medicamento y Productos Sanitarios (AEMPS). The study has been registered at ClinicalTrials.gov (identifier NCT02773173, May 16, 2016) and EudraCT (2016-001693-15), and is conducted in accordance with the Declaration of Helsinki on ethical principles for medical research in human subjects, adopted by the General Assembly of the World Medical Association (1996). Data management, monitoring and reporting of the study are performed in accordance with the International Conference on Harmonisation (ICH) Good Clinical Practice (GCP) guidelines (CPMP/ICH/135/95) and the regulatory requirements for participating institutions by the Spanish Clinical Research Network (SCReN). Investigators collect a written informed consent form in compliance with the GCP recommendations to the patient or his/her legal representative if the patient’s clinical conditions do not allow him/her to review and approve it. Investigators provide a copy of the signed informed consent form to each subject and keep a copy in the subject’s study file. This study protocol is reported following the Standard Protocol Items: Recommendations for Interventional Trials and Patient-Reported Outcomes (SPIRIT-PRO) guidelines [24, 25].

The results of the study will be communicated through the portal of the European Medicines Agency and will be sent for publication in a peer-reviewed medical journal. Authorship will be based on International Committee of Medical Journal Editors (ICMJE) criteria. No professional writer will be involved. After publication of the primary results, upon request, the pooled dataset will be available for all members of the IPPCollapse-II study group for secondary analysis, after judgement and approval of the scientific quality and validity of the proposed analysis by the Steering Committee. Access to source data will be made available through national or international anonymised datasets upon request and after agreement of the IPPCollapse-II Steering Committee.

## Discussion

This study is the first randomised clinical study that tests the hypothesis that an individualised pneumoperitoneum pressure strategy focusing on using the lowest possible IAP, compared to a conventional pneumoperitoneum pressure strategy, improves recovery after laparoscopic colorectal surgery. This study uses the PQRS as well as the occurrence of postoperative complications until postoperative day 28 and also hospital LOS. Furthermore, we assess process-related outcomes like IAP and IAV during pneumoperitoneum and associated ventilator parameters. A strong multidisciplinary commitment between members of the perioperative team, consisting of surgeons and anaesthesiologists, makes this complex study feasible.

The IPPCollapse-II study has several strengths. Its prospective design will allow high accuracy of data to be collected, and its sample size allows us to draw valid conclusions. Selection of PROs as the primary outcome of this study facilitates the translation into clinical practice, since these outcomes are readily and easily perceivable by both patients and healthcare providers. To the best of our knowledge, this is the first multicentre randomised clinical study evaluating the clinical effect of a tailored IAP management. The surgeon will remain blinded for the IAP, allowing us to titrate the IAP to the lowest possible level, i.e. the level at which surgeons have adequate workspace. Furthermore, we aim to describe the relationship between the IAP and actual IAV at which surgery is performed. This could lead, on the one hand, to gathering evidence towards establishing a volume threshold (e.g. the actual workspace) for colorectal laparoscopic surgery to replace the standard pressure threshold, and on the other, to describing the abdominal pressure-volume relationship in a first attempt to achieve something similar to our understanding of lung dynamics during ventilation. Additionally, we directly link the respiratory system and abdomen by assessing the IAP and respiratory driving pressure relationship. This could bring us a step further in achieving protective ventilation in the operating room.

The study proposed here differs from previous studies on this topic. Most studies so far have evaluated the individual components of the multifaceted strategy and are largely focused on surgical conditions and not patient-centred outcomes. Also, they generally find minor gains from abdominal prestretching or patient positioning optimisation and offer inconclusive results or marginally positive effects for the level of neuromuscular blockade [35–45]. Two studies find IAP titration useful in decreasing conventional IAP management, but they do not focus on clinical outcomes [46, 47].

To our knowledge, only one study so far focused on quality of recovery, using the Quality of Recovery 40 (QoR-40), a 40-item questionnaire on quality of recovery from anaesthesia [36]. This study, comparing surgery at low IAP (6 mmHg) versus standard IAP (12 mmHg) during laparoscopic donor nephrectomy under deep neuromuscular blockade, found no differences in QoR-40 score. Of note, in this study surgeons were not blinded for the IAP and in 25% of patients surgery had to be converted to the standard pressure, probably due to the surgeon’s learning curve. We recently performed the IPPCollapse-I study, in which we evaluated the feasibility of the intervention being tested in the present study [23]. The intervention was found to be safe and highly feasible and resulted in an acceptable workspace at low IAP in most patients. We did not look at patient outcomes in the preceding study.

The PQRS has been successfully tested in previous studies to evaluate differences in recovery [48–51]. We acknowledge that finding differences in PROs by PQRS modifying a single strategy in a high quality environment could be difficult [52–54]. In order to evaluate minor differences in recovery, mainly in laboratory data, we perform three substudies. Levels of biomarkers (neutrophil-lymphocyte ratio, C-reactive protein, interleukin-6 and procalcitonin) in the postoperative recovery period are linked to immunosuppression and postoperative complications [55, 56]. Untargeted metabolomic intraoperative analyses of blood samples and peritoneum biopsies allow us to depict differences between groups in the intraoperative setting and generate a hypothesis for new studies. PDR_ICG_ has been used successfully to evaluate hepatic perfusion in critically ill patients with intra-abdominal hypertension [28] and could reveal differences in hepatic perfusion during pneumoperitoneum in this study.

This study has limitations. We exclude ASA IV patients, who could benefit more from working with low IAP. Since we test a multifaceted strategy, it will remain uncertain which part of the strategy will have the largest impact. In fact, it could very well be that not all parts have the same magnitude of effect, and it is even possible that some parts have negligible  effects. Of note, reversal of neuromuscular blockade with sugammadex instead of neostigmine could improve PQRS recovery at T40 although not at POD1 or POD3. Surgeons, blinded for the actual IAP, will evaluate surgical conditions in a practical dichotomous manner as adequate or not, depending on whether any corrective action is needed. This way of measurement might make comparisons with other studies difficult, such as those using the Leiden-Surgical Rating Scale. The anaesthesiologists collecting the PQRS evaluations are not blinded for the assigned strategy, as they were present during the surgical procedure. However, the risk of bias will be very low, as the PQRS is a ‘patient-reported’ outcome, and the patients remain blinded at all times, including the time at which the PQRS score is collected. We calculated the sample size of our study on PQRS differences; thus, our sample could be underpowered for some secondary outcome that can potentially require a larger sample. In conclusion, the IPPCollapse-II study is designed to test if an individualised pneumoperitoneum pressure and optimised management versus conventional care will affect the outcome of patients undergoing colorectal laparoscopic surgery using relevant patient-centred outcomes.

### 3.1. Trial status

The Protocol Version is Version 1.0; June 7, 2016. A competent IRB, as detailed in the text, approved this version of the protocol. Recruitment began on February 1st, 2017. The expected date for recruitment completion is October–November 2018.

## Additional files

Additional file 1: Reporting SPIRIT checklist for protocol IPPCollapse-II. (DOCX 27 kb)

Additional file 2: Collaborating centres in the IPPCollapse-II study and expected number of patients recruited. (DOCX 12 kb)

Additional file 3: Enhanced Recovery Pathways Spanish guidelines summary. RICA (Intensive recovery in abdominal surgery) (DOCX 126 kb)

Additional file 4: Protocol for substudies document. (DOCX 24 kb)

## Abbreviations

ASA: American Society of Anesthesiologists
BMI: Body mass index
eCRF: Electronic case report form
GCP: Good Clinical Practice
IAP: Intra-abdominal pressure
IAV: Intra-abdominal volume
IPPCollapse: Individualized Pneumoperitoneum Pressure in Colorectal Laparoscopic Surgery versus Standard Therapy
IRB: Institutional Review Board
LOS: Length of (hospital) stay
PACU: Post anaesthesia care unit
PDR_ICG_: Plasma disappearance rate of indocyanine green
PEEP: Positive end-expiratory pressure
POD: Postoperative day
PQRS: Post-operative Quality of Recovery Scale
PRO: Patient-reported outcome
PTC: Post-tetanic count
SCReN: Spanish Clinical Research Network
SPIRIT: Standard Protocol Items: Recommendations for Interventional Trials
TOF: Train of four
UPLC: Ultra performance liquid chromatography
ΔP_rs_: Respiratory system driving pressure
