# Correction to: An individualised versus a conventional pneumoperitoneum pressure strategy during colorectal laparoscopic surgery: rationale and study protocol for a multicentre randomised clinical study

**DOI:** 10.1186/s13063-020-4055-3

**Published:** 2020-01-13

**Authors:** O. Diaz-Cambronero, G. Mazzinari, C. L. Errando, M. J. Schultz, B. Flor Lorente, N. García-Gregorio, M. Vila Montañés, Daniel Robles-Hernández, L. E. Olmedilla Arnal, A. Martín-De-Pablos, A. Marqués Marí, M. P. Argente Navarro, and for the IPPCollapse-II study group

**Affiliations:** 1 Department of Anaesthesiology, Hospital Universitari i Politècnic La Fe, Valencia, Spain; 2 Perioperative Medicine Research Group, Instituto de Investigación Sanitaria La Fe (IIS laFe), Avinguda de Fernando Abril Martorell, 106, 46026 Valencia, Spain; 3 SCReN-IIS La Fe, PT17/0017/0035, Spanish Clinical Research Network (SCReN), Valencia, Spain; 4 Department of Anaesthesiology, Hospital Universitari i Politecnic la Fe, Valencia, Spain; 5 Department of Anaesthesiology, Consorcio Hospital General Universitario de Valencia, Valencia, Spain; 6 Department of Intensive Care & Laboratory of Experimental Intensive Care and Anesthesiology (L·E·I·C·A), Academic Medical Center, Amsterdam, The Netherlands; 7 Mahidol Oxford Tropical Medicine Research Unit (MORU), Mahidol University, Bangkok, Thailand; 8 Department of Colorectal Surgery, Hospital Universitari i Politècnic La Fe, Valencia, Spain; 9 Department of Anaesthesiology, Hospital General Universitario de Castellón, Castellón, Spain; 10 Department of Anaesthesiology, Hospital General Universitario Gregorio Marañón, Madrid, Spain; 11 Department of Anaesthesiology, Hospital Universitario Virgen Macarena, Sevilla, Spain

**Correction to: Trials**

**https://doi.org/10.1186/s13063-019-3255-1**

After publication of our article [1] the authors have notified us that there are changes in the primary outcome and the statistical analysis plan of the study. These changes were made after the recruitment of participants and after approval by the Institutional Review Board, and registration at clinicaltrials.gov (study identifier), but before cleaning and closing of the database.

The Postoperative Quality of Recovery Scale (PQRS), an outcome used in the IPPCollapse II study, is a five–dimensional ordinal scale designed to estimate patients’ recovery in the postoperative period [2]. Each patient is scored at predefined time points and is classified as either ‘recovered’ if the score reaches at least the predetermined baseline score or ‘not recovered’ if otherwise. The five dimensions are then combined in an ‘overall score’ – a patient is classified as ‘overall recovered’ if ‘recovered’ in every domain and as ‘overall not recovered’ if ‘not recovered’ in any of the five domains.

Outcome variables that are repeatedly assessed over time in the same study patients are to be treated as ‘repeated measures’ or ‘longitudinal data’ [3]. Common statistical techniques applied on cross-sectional data assume independence between observations [4]. This crucial assumption is not fulfilled by ‘repeated measures’ or ‘longitudinal data’. Ignoring this correlation can lead to biased estimates, invalid P values and confidence intervals, as well as loss of statistical power [5, 6].

We incorrectly detailed how the PQRS score was to be analysed. We suggested to treat the scores at the four different time points as individual outcomes. From hindsight we feel that this approach does not consider the conceptual underlying model (i.e., between patients’ variability) and the temporal design. Furthermore, we also imperfectly reported our primary outcome since we did not specified which domain of the scale was analyzed as primary endpoint although we did report which one we used (i.e. physiologic score) in the sample size calculation. We therefore changed the primary and secondary outcomes as follows:

1. The primary outcome of the IPPCollapse II study is the recovery of the ‘physiologic’ component of the PQRS score over the assessed time points;
2. The other domains, i.e., the ‘nociceptive’, ‘emotional’, ‘cognitive’, and ‘functional’ components, as well as the ‘overall score’ are used as secondary outcomes;
3. Association between group assignment and recovery of PQRS score in each domain is assessed by a mixed logistic regression, introducing patients as random factors, and age, weight, BMI and sex as covariables;
4. The originally reported analysis (i.e. ordinal regression) is still carried out, however only as a sensitivity analysis.
