# Supplementary material for: An individualised versus a conventional pneumoperitoneum pressure strategy during colorectal laparoscopic surgery: rationale and study protocol for a multicentre randomised clinical study
Source: Trials. 2019 Apr 3;20:190. doi: 10.1186/s13063-019-3255-1 (PMC6446296; doi:10.1186/s13063-019-3255-1)
Supplement: Supplementary file 2 — Collaborating centres in the IPPCollapse-II study and expected number of patients recruited. (DOCX 12 kb) [file 13063_2019_3255_MOESM2_ESM.docx]

| **Additional file 2. Collaborating centres in the IPPCOLLAPSE II study, and expected number of patients recruited** | |
| --- | --- |
| **Hospital** | **Number of patients expected to be recruited (n)** |
| Hospital Universitari I Politecnic la Fe, *Valencia*, Spain | 100 |
| Hospital General Universitario, *Castellon*, Spain | 30 |
| Hospital General Universitario Gregorio Marañon, *Madrid*, Spain | 30 |
| Hospital Universitario Virgen Macarena, *Sevilla*, Spain | 30 |
