# Supplementary material for: An individualised versus a conventional pneumoperitoneum pressure strategy during colorectal laparoscopic surgery: rationale and study protocol for a multicentre randomised clinical study
Source: Trials. 2019 Apr 3;20:190. doi: 10.1186/s13063-019-3255-1 (PMC6446296; doi:10.1186/s13063-019-3255-1)
Supplement: Supplementary file 3 — Enhanced Recovery Pathways Spanish guidelines summary. RICA (Intensive recovery in abdominal surgery) (DOCX 126 kb) [file 13063_2019_3255_MOESM3_ESM.docx]

| **Additional file 3. Enhanced Recovery Pathways Spanish guidelines summary**  **RICA ( Intensive recovery in abdominal surgery)** | | | |
| --- | --- | --- | --- |
| **TIME** | **PROTOCOL** | **PROFESSIONAL** | |
| Before hospitalization | Preoperatory evaluation, nutritional optimization, cardiologic test if it is indicated. | | Surgeon  +  Anaesthesiologist |
| Immediate preoperative  (without previous hospitalization) | Thromboembolic prophylaxis  (12 h before surgery).  Preoperative solid fast 6 hours and clear liquid fast 2 hours.  In colon surgery is not indicated bowel mechanical preparation (reserve to rectum surgery) | | Surgeon  +  Anaesthesiologist  +  Nurse |
| Perioperative | **Previous surgery**  Cleaning enema at 7 am (colorectal surgery)  Compression tights or intermittent pneumatics socks for thromboembolic prophylaxis.  Carbohydrate 12,5% maltodextrins drinks 250cc 2 hours before surgery.  Antibiotic prophylaxis 1 hour before incision.  **Intraoperative**  Insertion epidural catheter in laparotomy surgery  Anaesthesia induction  Oxygenation FiO2 60-80%  Active warming with air convection blanket  Goal directed fluid therapy  Balanced solution (laparoscopy 3,5ml/Kg/h)  Minimal invasive surgery (when it is possible)  Avoid nasogastric tube  Nauseas and vomits prophylaxis using Apfel scale.  Local anaesthesia in laparoscopic access vs Abdominal Transverse Block.  Bladder catheter  **Immediate postoperative**  Maintenance active warming.  FiO2 50% at least 2 hours.  Restrictive fluid therapy  Oral tolerance 6 hours after surgery  Early mobilization  Thromboembolic prophylaxis with Enoxaparin 40mg at 10pm.  Reduce as minimal opioid administration | | Nurse    Nurse  +  Surgeon  +  Anaesthesiologist    Nurse  +  Surgeon  +  Anaesthesiologist |
| POD1 | Diet depends on tolerance  Active mobilisation (sitting)  Intravenous analgesia  Consider withdraw bladder catheter  Consider withdraw abdominal drains.  Nutritional supplements | | Nurse  +  Surgeon  +  Anaesthesiologist |
| POD2 | Normal diet  Active mobilisation (start ambulation)  Thromboembolic prophylaxis | | Nurse  +  Surgeon  +  Anaesthesiologist |
| Postoperative until hospital discharge | Normal diet  Oral analgesia  Active mobilisation Thromboembolic prophylaxis | | Nurse  +  Surgeon  +  Anaesthesiologist |
| Home | Thromboembolic prophylaxis until POD28  Phone contact  Ambulatory support | |  |

Abbreviations : POD: Postoperative day
