# Supplementary material for: An individualised versus a conventional pneumoperitoneum pressure strategy during colorectal laparoscopic surgery: rationale and study protocol for a multicentre randomised clinical study
Source: Trials. 2019 Apr 3;20:190. doi: 10.1186/s13063-019-3255-1 (PMC6446296; doi:10.1186/s13063-019-3255-1)
Supplement: Supplementary file 4 — Protocol for substudies document. (DOCX 24 kb) [file 13063_2019_3255_MOESM4_ESM.docx]

**An Individualized versus a Conventional Pneumoperitoneum Pressure Strategy during Colorectal Laparoscopic Surgery – rationale and study protocol for a multicentre randomised clinical study**

O. Diaz-Cambronero^1,2,3^, G. Mazzinari^2,4^, C.L. Errando^5^, M.J. Schultz^6,7^, B. Flor Lorente^8^, N. Garcia Gregorio^1,2^, M. Vila Montañes^1,2^, D. Robles Hernandez^9^, L.E. Olmedilla Arnal^10^, A. Martin de Pablos^11^, A. Marques Marí ^2^ and M.P. Argente Navarro^1,2^ for the IPPColLapSe II study group*

**Hospital Universitari i Politecnic La Fe, Valencia, Spain:**

^1^Department of Anaesthesiology

**Instituto de Investigación Sanitaria La Fe (IIS laFe):**

^2^Perioperative Medicine Research Group

**Spanish Clinical Research Network (SCReN):**

^3^SCReN-IIS La Fe, PT17/0017/0035

**Hospital de Manises, Valencia, Spain:**

^4^Department of Anaesthesiology

**Consorcio Hospital General Universitario de Valencia, Valencia, Spain:**

^5^Department of Anaesthesiology

**Academic Medical Center, Amsterdam, The Netherlands:**

^6^Department of Intensive Care & Laboratory of Experimental Intensive Care and Anesthesiology (L·E·I·C·A)

**Mahidol University, Bangkok, Thailand:**

^7^Mahidol Oxford Tropical Medicine Research Unit (MORU)

**Hospital Universitari i Politecnic La Fe, Valencia, Spain:**

^8^Department of Colorectal Surgery

**Hospital General Universitario de Castellón, Castellón, Spain:**

^9^Department of Anaesthesiology

**Hospital General Universitario Gregorio Marañón, Madrid, Spain:**

^10^Department of Anaesthesiology

**Hospital Universitario Virgen Macarena, Sevilla, Spain:**

^11^Department of Anaesthesiology

**Additional file 4. PROTOCOL DOCUMENT**

**1. Sample processing, preparation and analysis. Protocol for substudies of IPPCollapse–II.**

*Sample processing*

Blood samples for the level of biomarkers are collected according to usual clinical practice in each collaborating centre and analysed by its respective reference laboratory.

Blood samples for metabolomics analysis are collected prior to anesthesia induction, immediately after pneumoperitoneum generation and at the end of the laparoscopic procedure. Samples consisting of 5 ml of blood are extracted from a peripheral venous access in a heparin anticoagulant tube, and identified with the patient's identification number and sample number. Samples are kept at 4ºC before being transferred to the metabolomics unit within the hospital within 2 hours. The samples are centrifuged for 10 minutes at 1300 rpm and 4ºC. After centrifugation, 400uL plasma is aliquoted and stored at –80º C.

Peritoneal tissue samples for metabolomics analysis are collected following the same methodology at baseline after pneumoperitoneum generation and at the end of the laparoscopic procedure. Samples are identified with the patient's identification number and sample number and kept in liquid nitrogen tank located in the surgical unit until analysis.

*Sample preparation*

For the procedure of the plasma samples, once thawed, the proteins will be precipitated by using three volumes of organic solvent, centrifugation (3500 rpm), collecting the supernatant and transferring it to a chromatographic vial for analysis.

The treatment of the tissue samples will be carried out by homogenization with methanol in Precellys homogenizer at 4 ° C using two cycles of 25s at a speed of 6500rpm with intervals of 10 s. After centrifugation of the extract, the supernatant will be concentrated and redissolved in the ideal solution for subsequent chromatographic analysis.

LC-QToF Analysis

The metabolomics analysis will be carried out by means of a chromatographic separation using the UPLC (ultra performance liquid chromatography) chromatographic system available in the Analytical Unit and a Acquity UPLC HSS T3 type chromatographic column (100 x 2.1 mm, 1.8 μm) from Waters (Wexford, Ireland) or similar. The detection will be carried out by means of a mass spectrometer with time of flight analyser, 6550 QTOF Agilent, available in the Analytical Unit and ideal for "untargeted" approaches. The data in TOF MS full scan mode will be recorded from 50 to 1000 m / z (mass / load ratio) with a scan time of 0.1 s. A LockSpray interface will be used to maintain mass accuracy during the analysis.

The treatment of the samples, as well as the acquisition of data will be carried out under BPL regulations (good laboratory practices), which guarantees the quality and traceability of the results obtained.

*Data analysis*

The metabolomics comparative analysis between the different samples (data matrices) will require a processing of the data before its analysis, normally an alignment and a normalization. A chemometric approach will be applied, based on PACA and PLSDA models, for the selection of informative and discriminant variables (metabolites) that facilitate the marker selection process. Once the list of possible markers is configured, an unsupervised hierarchical analysis will be carried out in order to check their discriminatory capacity and subsequently they will be identified by consulting databases (HMDB, KEGG), MS / MS spectra and / or injection. of standards.

**2. Details on study logistics and data management**

*Study organization*

The principle investigator (Diaz-Cambronero) and the two investigators involved in the initial design of IPPColLapSe II study (Mazzinari and Errando) form the Steering Committee. Local main investigators are responsible for identifying and recruiting participating patients in each centre. They will assist and train local investigators and oversee conduct of the study, including administrative management, record keeping and data management. Local investigators at individual participating centres will provide scientific and structural leadership, ensuring local ethical and regulatory approvals are obtained before patient inclusion starts. The sponsor guarantees the quality and security of the data collected.

Prior to the start of the study, the teams in each centre will receive a training session on how to capture data in the electronic Case Report Form (eCRF). All team members will be provided with a manual of operations with instructions on how to accurately fill the forms and the screening log.

*Data management*

Data will be collected from the patient paper/electronic medical chart and recorded on paper CRF and successively transcribed into an electronic CRF (eCRF) at a later time point. Local investigators transcribe the collected data directly onto an anonymized internet–based eCRF (http://remote.iislafe.san.gva.es/ippcollapse/). Access to the data–entry system is protected by a personalized username and password. To optimize the quality of the data, the implemented eCRF automatically cross–check the entries and check for abnormal or erroneous values in data.

The data will be kept on a central secured server located at the Hospital Universitari i Politecnic la Fe, Valencia, Spain. Personal information will be protected as dictated by the Spanish Personal Data Protection Law (Ley Orgánica 15/1999 de Protección de Datos de Carácter Personal).

*Data monitoring*

Data managing, monitoring, and study reports will be done by independent monitors from the Spanish Clinical Research Network (SCReN; <https://www.scren.es>) as per the ICH-GCP Guidelines (CPMP/ICH/135/95). Monitoring activities will be conducted to ensure the protection of the rights and well–being of the participants in the clinical trial, to ensure that the data recorded are precise, complete and verifiable from the source documentation and that the conduct of the trial is done in accordance with the current approved version of the protocol and modifications in effect, with the GCPs, SOPs and any other applicable regulations. Sponsor’s monitors will guarantee that all parts involved in the trial receive training in the specific protocol procedures, that adverse events and follow–up are adequately reported, that the CRFs are completed on time, and that any major deviations from the protocol are identified and reported without delay. The frequency and proportion of parameter verifications will be performed at each centre in accordance with what is established in the Monitoring Plan. All monitoring activities, including initiation, follow–up and close out visits will be documented in accordance with the Sponsor’s procedures.
